# A sensitive electrochemical immunosensor for label-free detection of Zika-virus protein

**DOI:** 10.1038/s41598-018-28035-3

**Published:** 2018-06-26

**Authors:** Ajeet Kaushik, Adriana Yndart, Sanjeev Kumar, Rahul Dev Jayant, Arti Vashist, Ashley N. Brown, Chen-Zhong Li, Madhavan Nair

**Affiliations:** 1 Center of Personalized Nanomedicine, Institute of NeuroImmune Pharmacology, Department of Immunology, Herbert Wertheim College of Medicine, Florida International University, Miami, Florida 33199 USA; 2 Biomedical Instrumentation, CSIR-Central Scientific Instruments Organization, Sector 30-C, Chandigarh, 160030 India; 3 Institute for Therapeutic Innovation, Department of Medicine, College of Medicine, University of Florida, Orlando, Florida 32827 USA; 4 Nanobioengineering/Bioelectronics Laboratory, Department of Biomedical Engineering, Florida International University, 10555 West Flagler Street, Miami, Florida 33174 USA

## Abstract

This work, as a proof of principle, presents a sensitive and selective electrochemical immunosensor for Zika-virus (ZIKV)-protein detection using a functionalized interdigitated micro-electrode of gold (IDE-Au) array. A miniaturized IDE-Au immunosensing chip was prepared via immobilization of ZIKV specific envelop protein antibody (Zev-Abs) onto dithiobis(succinimidyl propionate) i.e., (DTSP) functionalized IDE-Au (electrode gap/width of 10 µm). Electrochemical impedance spectroscopy (EIS) was performed to measure the electrical response of developed sensing chip as a function of ZIKV-protein concentrations. The results of EIS studies confirmed that sensing chip detected ZIKV-protein selectively and exhibited a detection range from 10 pM to 1 nM and a detection limit of 10 pM along with a high sensitivity of 12 kΩM^−1^. Such developed ZIKV immune-sensing chip can be integrated with a miniaturized potentiostat (MP)-interfaced with a smartphone for rapid ZIKV-infection detection required for early stage diagnostics at point-of-care application.

## Introduction

Zika-virus (ZIKV) infection, a mosquito-borne viral infection associated with microcephaly and neuro-disorders, became a very serious global health concern^1,2^. ZIKV have modified itself in humans and can be maintained in large populations through a mosquito-human-mosquito transmission cycle where a non-human reservoirs is  not mandatory for transmission. The similarity between the symptoms caused by ZIKV and other flavivirus-infection disease made selective ZIKV diseases diagnostics very challenging and causes delay in therapy decision. The available ZIKV diagnostics methods such as ELISA and PCR are effective but they are very expensive, time consuming and expert labor intensive. Beside this, unavailability of effective vaccines and drugs raised the demand of early stage ZIKV detection needed for rapid diagnostics and infection progression monitoring to decide therapy timely^1,3^.

Serious efforts are being made to develop efficient analytical methods for rapid and selective qualitative assessment of ZIKV-infection in real samples at epidemic site-location. These approaches were useful for the screening of ZIKV infection progression and therapy efficacy assessment^1^. Recently a paper based biosensor fabricated using CRISPR-Cas9 (clustered regularly interspacedshort palindromic repeats-associated protein-9 nuclease) was developed to detect ZIKV-RNA genome^4,5^. A sensing system, proposed for point-of-care (POC) application, based on reverse transcription-loopmediated isothermal amplification (RT-LAMP) was developed for a sensitive detection of ZIKV^6^. Very recently, a smartphone based approach which function on RT-LAMP phenomena was proposed to detect ZIKV, chikungunya, and dengue viruses^7^. These methods are colorimetric and showed significant qualitative sensing outcomes. RNA based capacitive sensing methodologies^8^ and nanoparticles-assisted plamonic platform have also been explored for the quantitative estimation of ZIKV^9–11^. However, these methods are multi-component based systems and investigators of above discussed research suggested lot of optimizations followed by validations prior promote these methods for clinical application. Thus, least components based analytical tools for rapid diagnostics of ZIKV-infection at early stage are urgently required.

**Figure 1:**
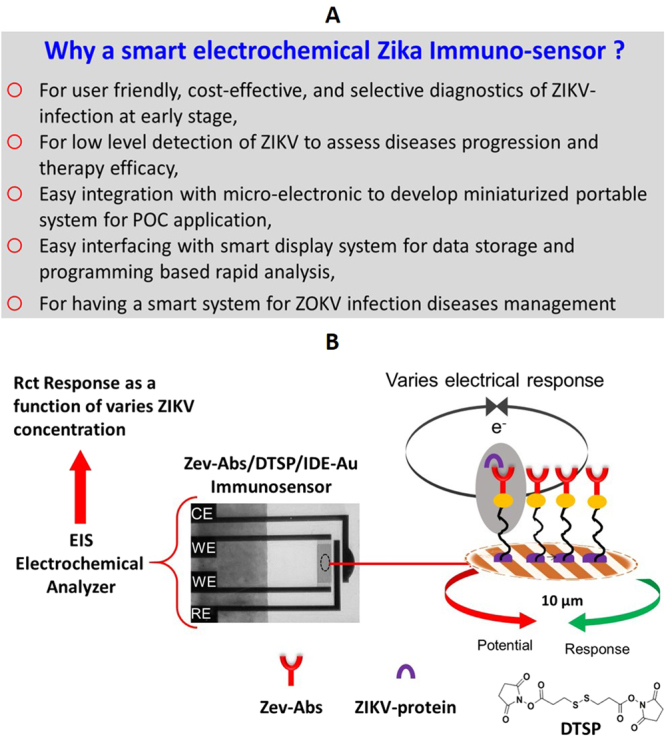
(**A**) Necessity of a smart sensitive ZIKV biosensor in order to manage ZIKV diseases, (**B**) Illustration of IDE-Au based electrochemical ZIKV immunosensor.

Aiming to develop an ASSURED i.e., affordable, sensitive, specific, user-friendly, rapid, robust, equipment free, and deliverable systems for ZIKV infection diseases, an electrochemical sensing system with the ability to detect a targeted analyte at low level could be one of best recommended approach^3,12–14^. For developing such systems, the introduction of nano/micro-electronics to develop miniaturized potentiostat (MP), nano/micro electrodes to develop sensing chips, nanostructures to load more amount of biomolecule, and automated BioMEMS functions at low volume has revolutionized biomarker detection at pM/fM needed for early stage diseases diagnostics^12–16^. The successful integration of these device components will resulted in smart sensing systems able to perform diagnostics at POC application. Recently, a graphene based nucleic acid based immunosensor was developed for the electrochemical detection of ZIKV. This biosensor functioned on field effect transistor (FET) based biosensing principle and detected ZIKV at 450 pM^17^. This sensor performed quantitative detection of ZIKV but at very high dose and may not be effective to detect ZIKV infection progression where the virus level variation is at pM level. For example, the virus infection level varies at very low level under a therapy. Thus developing systems with the ability to detect low ZIKV concentration (pM/fM) are urgently required for ZIKV diagnostics and therapy monitoring, as illustrated in Fig. 1A.

In this research, we present an electrochemical immunosensor (illustrated in Fig. 1B) fabricated via immobilization of ZIKV-envelop protein antibody (Zev-Abs) onto self-assembled monolayer (SAM) of DTSP deposited on IDE-Au for the selective detection of ZIKV concentration ranging from 10 pM to 1 nM using electrochemical impedance spectroscopy (EIS). The Zev protein was selected as a model target protein because this segment of ZIKV stricture bind with antibody with high affinity resulting in neutralization of ZIKV strain^18,19^. Our developed sensor exhibited detection limit of 10 pM and has potential to integrate with a MP-interfaced with smartphone for data storage and data analysis at POC. Thus generated bio-informatics can be useful to rapid diseases diagnostics and timely therapy decision.

## Results and Discussion

### Electrochemical characterization of sensor fabrication

**Figure 2:**
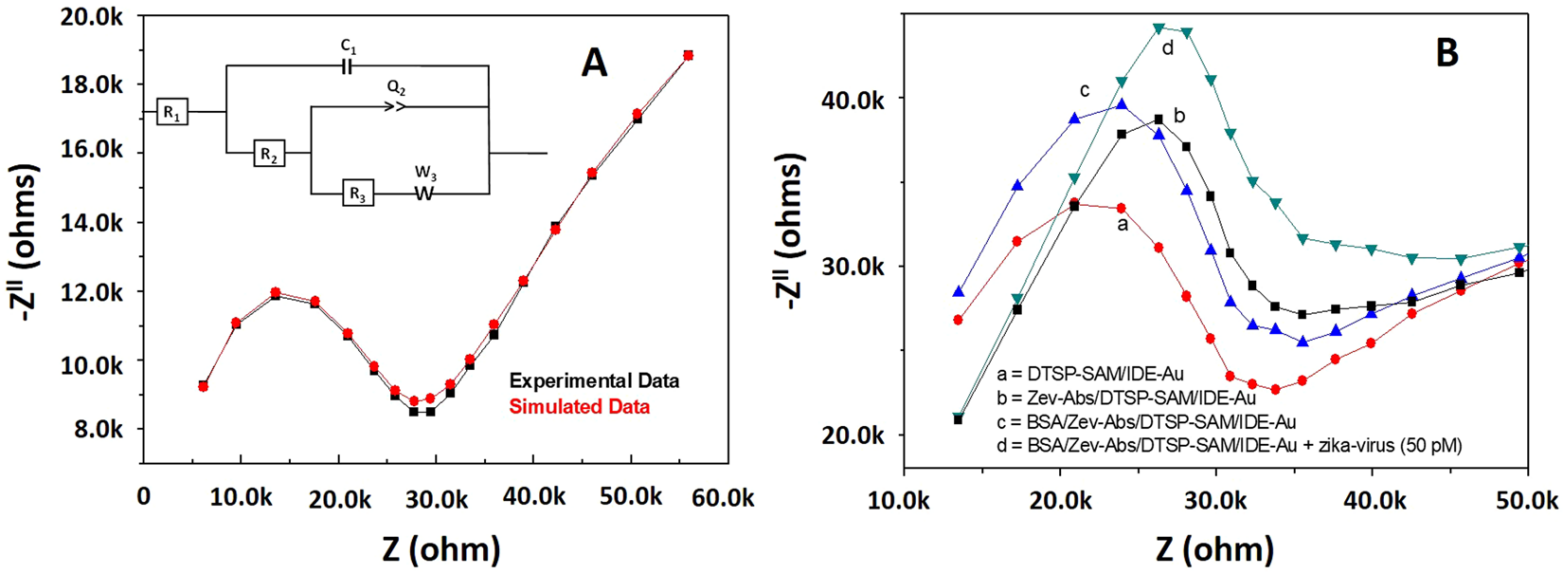
(**A**) EIS response of IDE-Au electrode and its electronic circuit simulation, (**B**) EIS studies of (a) DTSP modified IDEs-Au, (b) ZVev-Abs immobilized DTSP/IDEs-Au electrode, (c) BSA immobilized ZVev-Abs/IDEs-Au immuno-electrode, and (d) change is electrochemical response of immuno-electrode on adding 50 pM ZIKV using 5 µL of 5 mM PBS containing 5 mM Fe(II)/Fe(III) as redox moieties (pH = 7.4) at 100 kHz.

The IDE-Au exhibited a typical EIS Nyquist plot (black curve) as shown in Fig. 2A. Experimentally obtained data values were well-fitted with a simulated data (red curve) obtained from a circuit (inset, Fig. 2A). The semicircle of the Nyquist plot present charge transfer resistance (Rct) of the system which is very much sensitive to a very small changes occurs at the interface of electrode and electrolyte. The results of the EIS studies (Fig. 2B, curve a) showed that the Rct value of the DTSP/IDE-Au electrode increases after immobilizing Zev-Abs antibody. The covalent binding of Zev-Abs with DTSP make surface more insulating resulting in electron transfer hindrance led to high Rct value. This increment in Rct value confirmed the immobilization of Zev-Abs onto DTSP-SAM/IDE-Au electrode (Fig. 2B, curve b). The diameter of semicircle with reference to Zev-Abs/DTSP/IDE-Au immune-electrode was further increased after the immobilization of BSA protein. This observed increment in Rct value was due to insulating nature of BSA and confirmed the blocking of non-binding sites of the immuno-sensing chip surface (Fig. 2B, curve c). The electrochemical response of immuno-electrode was measured by adding 50 pM ZIKV i.e., ZIKV-envelop protein, (10 µL), for 30 minutes. The observed increment in Rct value (Fig. 2B, curve d) due to the formation of antigen-antibody complex confirming that sensor is responsive to ZIKV.

### Electrochemical immune-sensing of ZIKV protein

**Figure 3:**
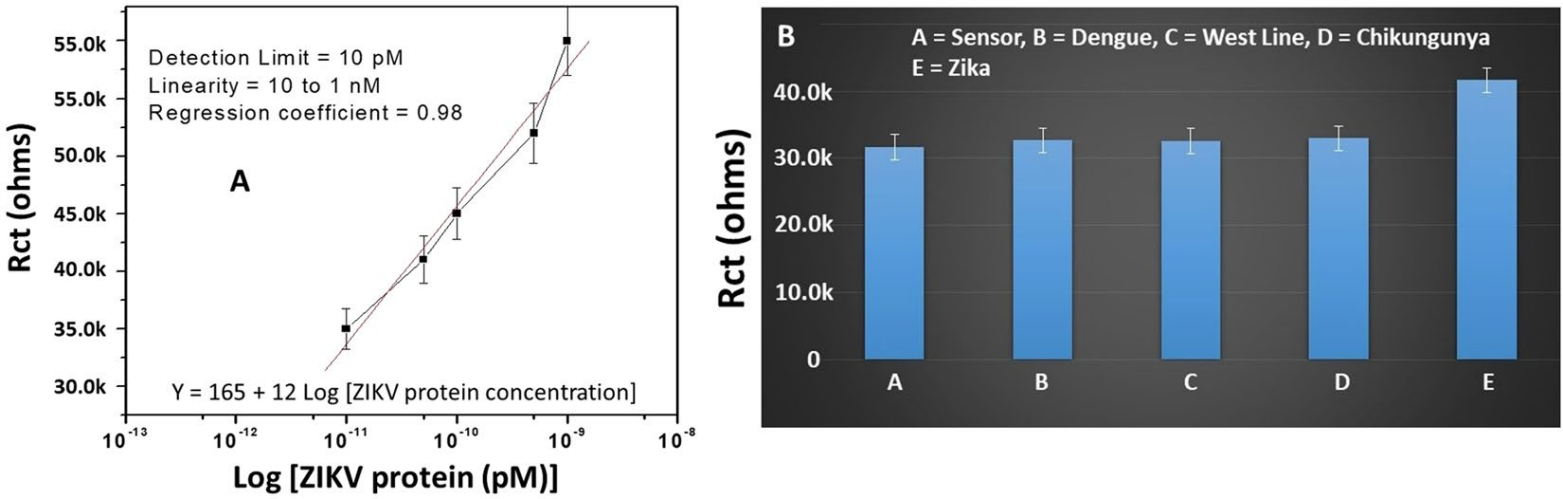
(**A**) Electrochemical response studies of developed immuno-sensor as a function of known ZIKV concentrations (10 pM to 1 nM). (**B**) Selectivity evaluation of developed ZIKV immunosensor with reference to interferents (10 µL of 50 pM) such as dengue, chikungunya, and west nile virus proteins.

The sensing performance of BSA/Zev-Abs/DTSP/IDEs-Au immuno-sensor was studied as a function of various ZIKV protein concentrations ranging from 10 pM to 1 nM using EIS in 5 µL of 5 mM PBS containing 5 mM Fe(II)/Fe(III) as redox moieties (pH = 7.4) at 100 kHz. An increment in Rct value was observed on adding each ZIKV protein concentration with reference to electrical response of chip without ZIKV protein. During the electrochemical ZIKV immuno-sensing, an antibody and antigen immuno-complex formed due to binding of ZIKV envelop antibody with ZIKV protein led to electron transport hindrance between medium to electrode. On increasing ZIKV protein concentration on sensing chip, the higher ZIKV protein adsorption led to obstruct the electrical response at the interface resulting in increased Rct magnitude. An incubation time of 30 minutes was optimized to achieve maximum binding between Zev-Abs and ZIKV envelop protein. A calibration curve [y = 165 + 12 × log (ZIKV concentration)] was plotted between obtained Rct values and ZIKV protein concentration (Fig. 3A). This linear calibration cure with the regression coefficient of 0.98 will be used to detect unknown concentrations of ZIKV in real samples. BSA/Zev-Abs/DTSP/IDEs-Au immuno-sensor exhibited a low detection limit of 10 pM, a linear detection range from 10 pM to 1 nM, and a high sensitivity of 12 kΩM^−1^.

### Stability and selectivity of developed electrochemical ZIKV immunosensor

To evaluate the selectivity of sensor, EIS response of our developed ZIKV immunosensing chip was tested in the presence of 10 µL envelop proteins of potential interferents viruses such as dengue virus (50 pM), chikungunya virus (50 pM), and Nile West virus (50 pM), causes fever similar to ZIKV symptoms. The results of EIS studies confirmed that BSA/Zev-Abs/DTSP/IDEs-Au immuno-sensor showed no effect (2–3% variation in Rct value) of interferents and was exhibited response only after adding ZIKV (50 pM) protein (Fig. 3B). These outcomes suggested that immuno-sensor has negligible effect of interferents and selected Zev antibody is selective to the ZIKV only. An EIS study was also carried out to study the shelf-life (data not shown) of the BSA/ZV-Abs/DTSP/IDEs-Au immuno-sensor at intervals of 1 week. Obtained results confirm that the sensor is stable for 30 days at 4 °C and beyond that time the magnitude of electrochemical response reduced significantly.

**Figure 4:**
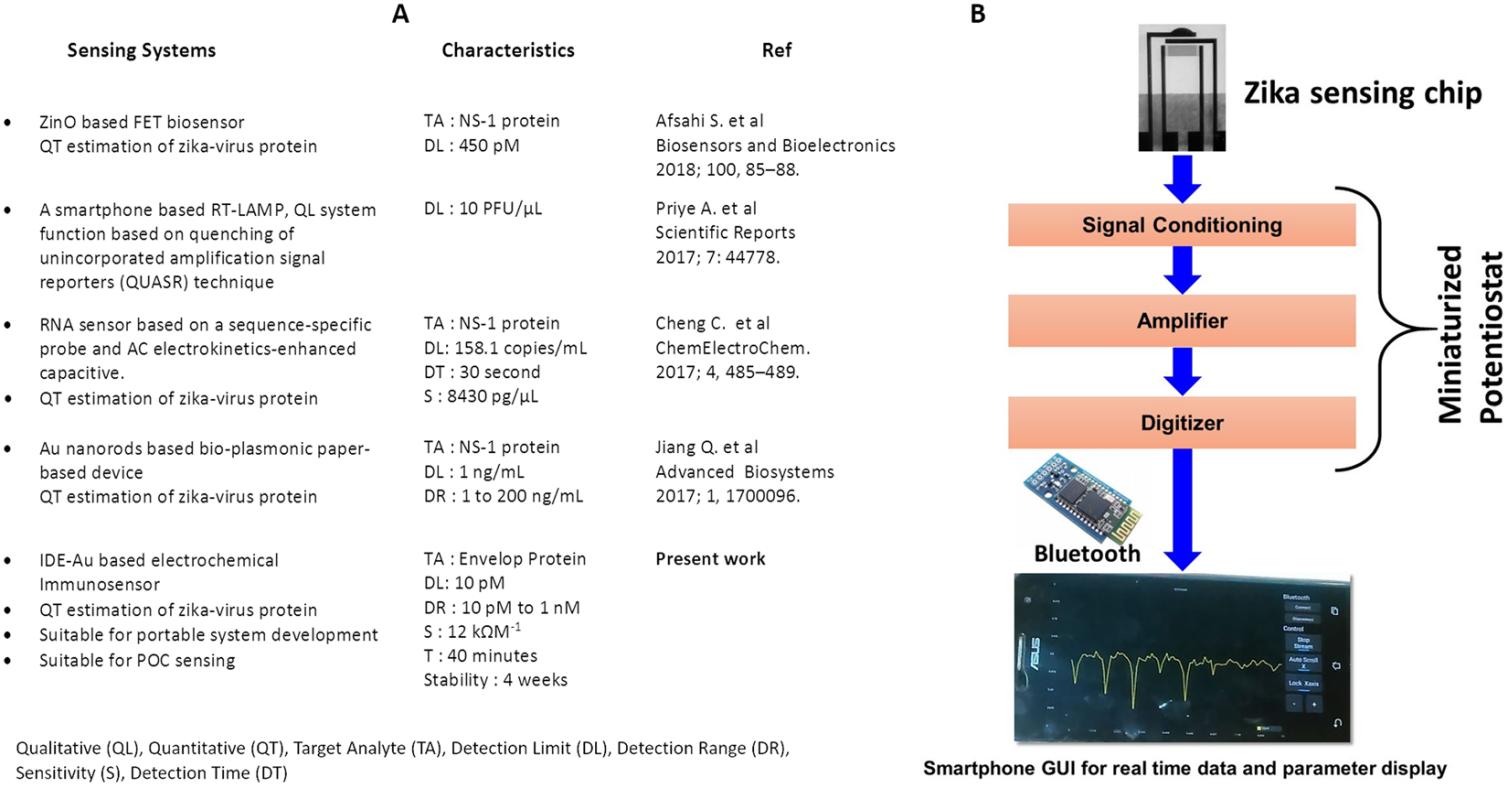
(**A**) Summary of IDE-Au based electrochemical ZIKV immunosensor characteristics in caparison of literature. (**B**) Roadmap of future plan in order to develop an electrochemical ZIKV immunosensor at POC application.

As viewpoint, thus developed micro-electrochemical ZIKV immunosensor is able to detect ZIKV protein selectively at pM. Such of sensitive system is required for the early stage ZIKV infection diagnostics, assessment of therapy efficacy and generating data with it clinical relevance to manage/understand epidemic. Beside this, and development of POC system to make ZIKV diagnosis easy at every location will certainly be useful to manage ZIKV infection related diseases. Our developed sensor exhibited improved performance in comparison of reported in literature as summarized in Fig. 4A. In future, our aim is to detect ZIKV at POC (as illustrated in Fig. 4B) after optimizing all the device components i.e., sensing chip (presented in this manuscript), integration of chip with MP (in progress), interfacing of analytical unit with smart display system (in progress), and finally ZIKV sensing using smartphone based operation. Overall, proposed smartphone based sensing system will serve as an analytical tool for ZIKV infection diseases management via rapid ZIKV diagnostics needed to monitor infection progress and therapeutic efficacy assessment. However, herein presented sensor is not yet tested using real samples due to lack of administrative formalities. Serious efforts are being made to establish a collaboration with Florida based hospitals to arrange bio-fluids of ZIKV infected patient. Moreover, the formalities of Institutional Biosafety committee (IBC) and Institutional Review Board (IRB) approval have also been initiated to have institutional permission to conduct research related with further optimization and validation of sample. The results of the complete future studies related with POC sensing of ZIKV will be published elsewhere.

## Conclusion

Aiming to develop an analytical tool for early stage diagnostics of ZIKZ-infection diseases, we fabricated an impedimetric micro-immunosensor using Zev-Abs for the selective detection of ZIKV-protein. The rapid (operation time around 40 minutes) and selective detection of ZIKV in wide concentration range with a low detection limit (10 pM) is certainly of clinical use for ZIKV diseases monitoring, therapy assessment, and timely therapy decision. As future direction, efforts are being made to detect ZIKV in real samples and to validate performance using ELISA/PCR standard methods. Thus developed sensing chip can be promoted for ZIKV sensing at POC for personalized health care management.

## Methods

The IDE-Au array (chamber volume ∼5 μL, electrode width and electrode gap 10 μm), function on three-electrode based electrochemical measurement, were procured from Micrux Technologies. The DTSP, sodium borohydride (NaBH4), and bovine serum albumin (BSA) were purchased from Sigma-Aldrich and were used without any further purification. ZIKV-envelop protein antibody (AZ1176) and related monoclonal antibody (Zev-Abs) were preceded from Aalto Bio Reagent and Sino Biological Inc., respectively. The interferents proteins such as dengue type 2, envelope (N-Term) protein (Product No. ABIN572971), chikungunya E2 (His tag) protein (Product No. ABIN1605333), and west nile virus envelope protein (N-Term) Protein (Product No. ABIN573053) were procured from antibodies-online.com. Phosphate buffer saline (PBS) solution (10 mM, pH 7.4) was prepared by dissolving one PBS tablet in 200 mL of de-ionized (DI) water. The PBS (pH 7.4) water was used to prepare stock solutions of Zev-Abs (1 mg/mL) and ZIKV envelop protein (1 mg/mL) and both the solutions were stored at −20 °C for future experiment.

An electrochemical ZIKV immunosensor was fabricated using IDE-Au to achieve ZIKV detection at pM level. Our established procedure was adopted for the electrochemical cleaning of IDE-Au and functionalization of IDE-Au by DTSP^20^. In brief, electrochemically cleaned IDEs-Au were dispersed in 2 mg/mL solution of DTSP for 2 hrs to prepare antibody immobilizing platform. This matrix was washed with DI water to remove unbind particles and dried at 4 °C^21^. Specific Zev-Abs monoclonal antibody (10 µl of 1 mg/mL of Zev-Abs, specific to ZIKV envelop protein) was immobilized via electrostatic interactions onto DTSP/IDEs-Au for 2 hrs to detect ZIKV selectively. The non-binding surface of Zev-Abs/SAM/IDEs-Au electrodes were blocked using BSA protein. Finally, the immuno-electrodes were washed using PBS (pH 7.4, 10 mM) to remove unbind molecules. All fabricated sensing chips i.e., BSA/Zev-Abs/SAM/IDE-Au immuno-electrodes were stored in the refrigerator at 4 °C when not in use. The schematics of proposed sensor fabrication and working principle is shown in Fig. 1B. The stepwise characterization of immunosensor fabrication was performed using EIS (multi-channel potentiostat/galvanostats of BioLogic instruments), as a function of charge transfer resistance (Rct) parameter, in 5 mM PBS containing 5 mM Fe(II)/Fe(III) as redox moieties (pH = 7.4).

The sensing performance of developed ZIKV immunosensor was performed at room temperature using EIS (in terms of Rct magnitude) as a function of various ZIKV concentration ranging from (10 pm to 1 nM) in 5 mM PBS containing 5 mM Fe(II)/Fe(III) as redox moieties (pH = 7.4). Prior to perform sensing, 10 µL of each ZIKV-protein concentration was incubated for 30 minutes onto sensing chip to achieve maximum binding of antibody-protein. After immuno-complex formation, the immuno-electrode chip was washed with DI water to remove unbounded protein from the surface.
